# Implementation of a single-port robotic platform into your colorectal surgery practice: a SAGES colorectal committee task force review of evolving robotic technology and innovations

**DOI:** 10.1007/s00464-026-13471-4

**Published:** 2026-10-12

**Authors:** Ankit Sarin, Justin Dourado, Christopher M. Seffren, Upinder S. Sidhu, Poya Pourghaderi, Nathan Aminpour, Elisabeth C. McLemore, Giovanni Dapri, Nawar A. Alkhamesi, Sherief Shawki, Brooklyn Williams, Muhammed Elhadi, Scott Nguyen, Alison Wenholz, Patricia Sylla, Laila Rashidi

**Affiliations:** 1 Department of Surgery, UC Davis Medical Center, 6th Floor, 2335 Stockton Blvd., Sacramento, CA 95817 USA; 2 Department of Surgery, Florida Atlantic University, Boca Raton, FL USA; 3 Department of Surgery, UC Davis Medical Center, Sacramento, CA USA; 4 School of Medicine, University of Arizona, Phoenix, AZ USA; 5 Department of Colorectal Surgery, MultiCare Cancer Institute, Tacoma, WA USA; 6 Department of Surgery, Los Angeles Medical Center, Kaiser Permanente, Los Angeles, CA USA; 7 International School Reduced Scar Laparoscopy, University of Mons, Mons, Belgium; 8 Minimally Invasive General and Oncologic Surgery, Humanitas Gavazzeni and Castelli University Hospital, Bergamo, Italy; 9 Kadlec Regional Medical Center and Elison S. Floyed College of Medicine, Washington State University, Richland, WA USA; 10 Department of Surgery, Mayo Clinic, Rochester, MN USA; 11 Department of Surgery, Army Medical Center, Tacoma, WA USA; 12 Department of Surgery Division of Colon and Rectal Surgery, Houston Methodist Hospital, Houston, TX USA; 13 Department of Colorectal Surgery, MultiCare Cancer Institute, Tacoma, WA USA; 14 Department of Surgery, Mount Sinai Health System, New York, NY USA

**Keywords:** Single-port robotic surgery, Colorectal surgery, Robotic surgery, Minimally invasive surgery, Robotic surgery platforms

## Abstract

**Background:**

Robotic and endoscopic techniques continue to revolutionize minimally invasive surgery. Single-port (SP) robotic platforms mark a new era in minimally invasive colorectal surgery, combining single-incision/site access with full robotic articulation through a single access point. This manuscript, as a part of a four-part series, outlines specific technical considerations of the SP robotic platform including details of the platform, instrumentation, docking, and operative maneuvers.

**Methods:**

Subject matter experts from the SAGES Colorectal Committee SP Task Force performed a structured review of the currently FDA-approved SP robotic platform, assessing technical features, instrumentation, and workflow considerations for several key representative operations relevant to colorectal surgery. Three clinical areas of SP implementation included transanal robotic surgery, right colectomy, and low anterior resection (LAR). Task Force members (subject matter experts with early experience in SP robotic platforms) analyzed technical details, patient selection, and operative workflows. Representative procedural techniques were detailed with emphasis on instrumentation choice, docking strategies, and key operative steps specific to single-port colorectal surgery.

**Results:**

The technical aspects of the SP platform include full robotic articulation through a single 25 mm port. A SP approach also enables triangulation and dexterity within confined spaces using three wristed instruments and a flexible 3-D camera. The current 360-degree boom rotation facilitates multi-quadrant manipulation without redocking. Current limitations may include continued reliance on bedside assistance for certain energy and stapling operative steps, although stapling capability has expanded with FDA clearance of an SP-compatible 45 mm stapler as of April 2025. Training of the entire operative team and gradual progression of case complexity is recommended as the surgeon implements this new technology into clinical practice.

**Conclusions:**

A SP robotic approach to minimally invasive colorectal surgical procedures is feasible for a range of colorectal surgery operations. In select procedures like SP TAMIS, SP right colectomy, and SP-LAR, the single-port platform appears to offer advantages related to reduced invasiveness and improved surgeon ergonomics compared to other minimally invasive approaches. Successful implementation requires structured training, careful patient selection, and adherence to evidence-based adoption principles.

Currently, there is only one FDA-approved single-port (SP) robotic platform that integrates single-incision access with advanced robotic dexterity, maintaining triangulation and precision through a single 25 mm port [1, 2]. The SP platform expanded to colorectal surgery with specific approval in 2024 [3]. Its configuration—three fully articulating instruments and a flexible 3-D camera within a multichannel cannula—can overcome limitations of earlier single-incision techniques such as instrument collisions and surgeon stance and ergonomics while preserving the benefits of reduced abdominal wall trauma and improved cosmesis [4].

The shift from open to laparoscopic techniques reduced postoperative pain, hospital stay, and recovery time without compromising oncologic outcomes [5]. Early multiport robotic colorectal surgery demonstrated technical feasibility and ergonomic advantages over conventional laparoscopy, establishing a foundation for subsequent oncologic and reconstructive advances [6]. Robotic platforms subsequently facilitated widespread adoption of intracorporeal anastomosis in colorectal surgery, permitting greater flexibility in extraction site, which may reduce anatomic localization extraction-site morbidity of midline incisions compared with extracorporeal anastomosis (with right colectomy being most dramatically impacted) [7]. However, both laparoscopic and multiport approaches require multiple incisions, each carrying risks of port-site complications, herniation, and potential cosmetic concerns [8] for patients.

Single-incision laparoscopic surgery (SILS) sought to minimize trauma further but was limited by loss of triangulation, instrument crowding, and surgeon fatigue [9]. The SP platform directly addresses these constraints through 360-degree boom rotation for full multi-quadrant access and articulating instruments that maintain triangulation through a single incision [10]. By consolidating access to a single incision, SP approaches may reduce cumulative abdominal wall trauma, although definitive comparative data on wound morbidity remain limited [10].

Robotic platforms are constantly evolving [11] and were adapted for transanal surgery in 2012, improving dexterity and visualization, but posing challenges with complex setup and external arm collisions [12]. The SP platform addresses these issues through flexible wristed instrumentation that maintains natural hand–eye coordination, and an articulating “cobra” camera that provides stable visualization of the rectal walls without repeated repositioning [13]. The third robotic arm enables retraction and facilitates advanced maneuvers such as full-thickness excision and precise suture closure [14].

This manuscript details the important technical elements of the SP platform as well as training and implementation considerations. Three additional parts in the series will further describe the application including video demonstrations of the single-port platform in three common colorectal procedures: SP transanal minimally invasive surgery (TAMIS), SP right colectomy, and SP low anterior resection (LAR)/total mesorectal excision (TME).

## Methods

Subject matter experts (SMEs) of the SAGES Colorectal Committee SP Task Force conducted a structured review of the currently FDA-approved SP platform, focusing on key platform features, available instrumentation, and workflow considerations specific to TAMIS, right colectomy, and LAR/TME. The SMEs consisted of colorectal surgeons with extensive experience in laparoscopic and multiport robotic colorectal procedures who had early experience with SP robotic platforms.

The task force reviewed the published literature on use of SP platforms in colorectal surgery and discussed this in the context of the collective SME experience. Technical considerations including incision placement, docking strategies and tools, and procedural techniques focused on exposure, dissection, vascular control, anastomosis (or defect closure), and other important operative steps. Patient selection criteria and operative workflows/steps were organized through review of successful techniques across published work and SME experience. Early clinical outcomes from published series and cadaveric studies were considered in developing surgeon and institutional recommendations for implementation of a SP platform in colorectal surgery procedures.

This series of manuscripts was developed through iterative review by all task force members to ensure accuracy and clinical applicability. Following manuscript completion, an artificial intelligence-based language editing tool was employed to enhance grammatical accuracy, clarity, and overall readability. No substantive modifications were made to the scientific content or interpretation of data.

## Results

### Single-port robotic platform overview

**Fig. 1:**
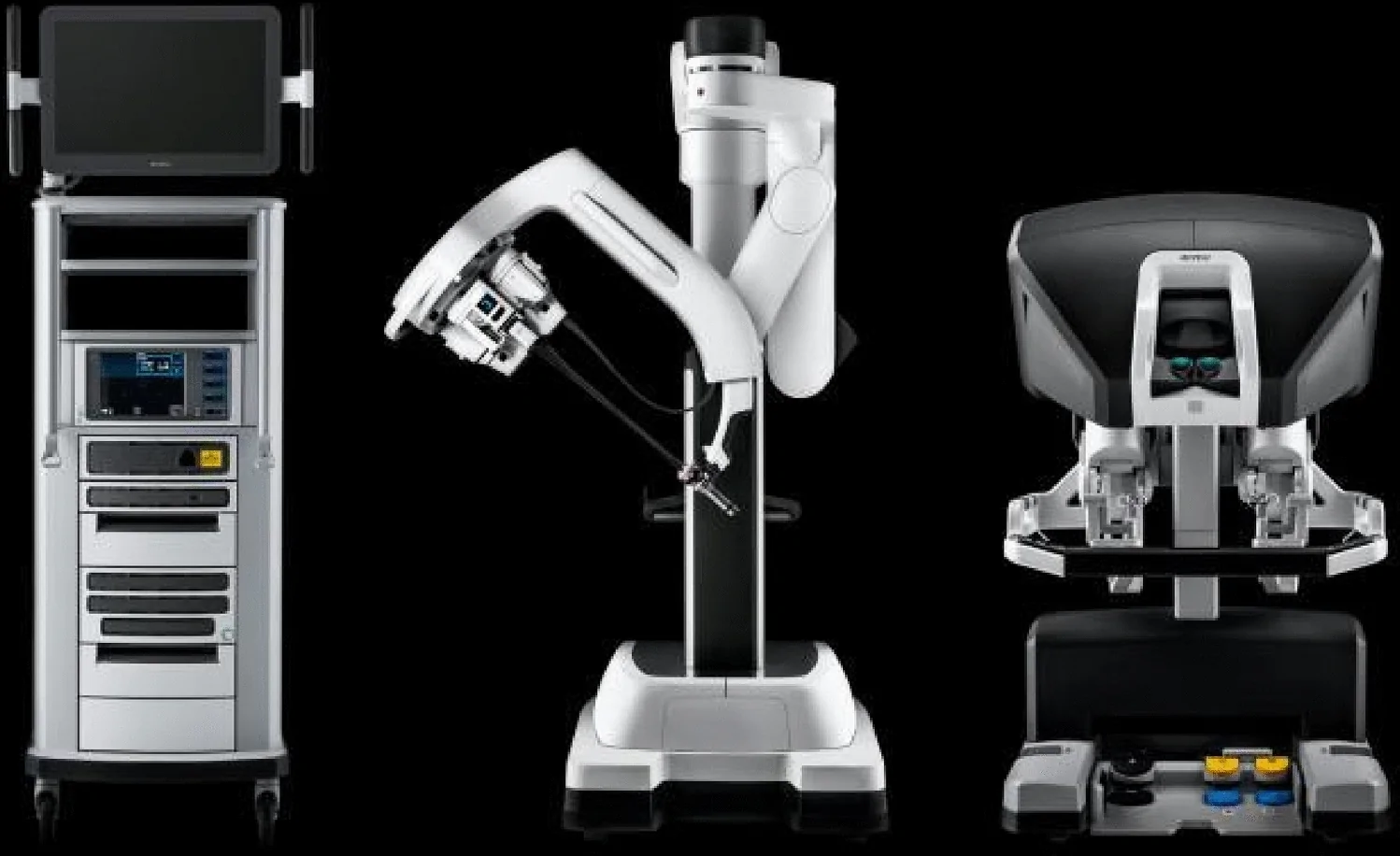
Three main components of the da Vinci SP platform vision cart, patient cart with robotic arm, and surgeon console

**Fig. 2:**
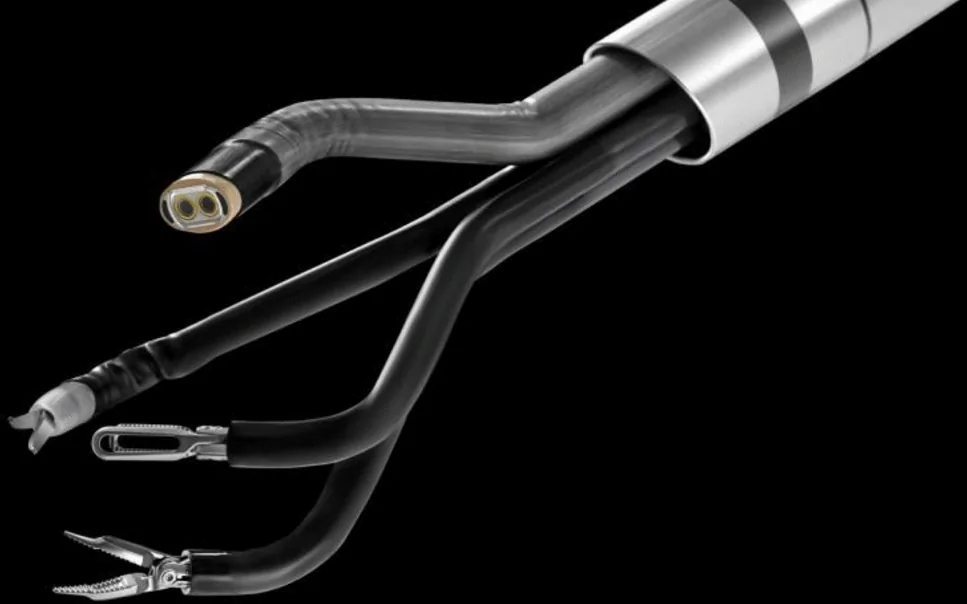
Robotic arm with three working instruments and one flexible tip camera

The currently FDA-approved SP robotic platform consists of three main components: a patient cart with a single robotic arm, a surgeon console, and a vision cart [15] (Fig. 1). The SP platform utilizes a single robotic arm accommodating four channels within a 25 mm cannula: three for working instruments and one for a camera (Fig. 2).

The single-arm design eliminates multiport docking while maintaining instrument triangulation through a novel cannula configuration [16]. The platform boom rotates 360-degrees around the port’s remote center, enabling multi-quadrant access without repositioning, which is advantageous for complex colorectal procedures requiring extensive colonic mobilization. Alternatively, this feature has limited application in transanal procedures where the operative field remains fixed relative to intra-abdominal procedures.

The SP cannula is designed for insertion through a 27 mm incision using standard open technique. The cannula assembly includes the SP cannula, Entry GuideTM cannula seal, and SP obturator, which must be properly assembled prior to insertion [17]. The cannula features integrated insufflation capabilities and maintains pneumoperitoneum while accommodating multiple instruments. Proper alignment of the remote center with the patient’s body wall is essential for optimal instrument reach throughout the procedure. The four-channel design of the arm allows simultaneous deployment of three articulated instruments and a flexible endoscope. Early clinical series suggests that acceptable operative times and workflow efficiency can be achieved with the SP platform following the learning curve, though direct equivalence with multiport robotic surgery has not been conclusively established [18].

**Fig. 3:**
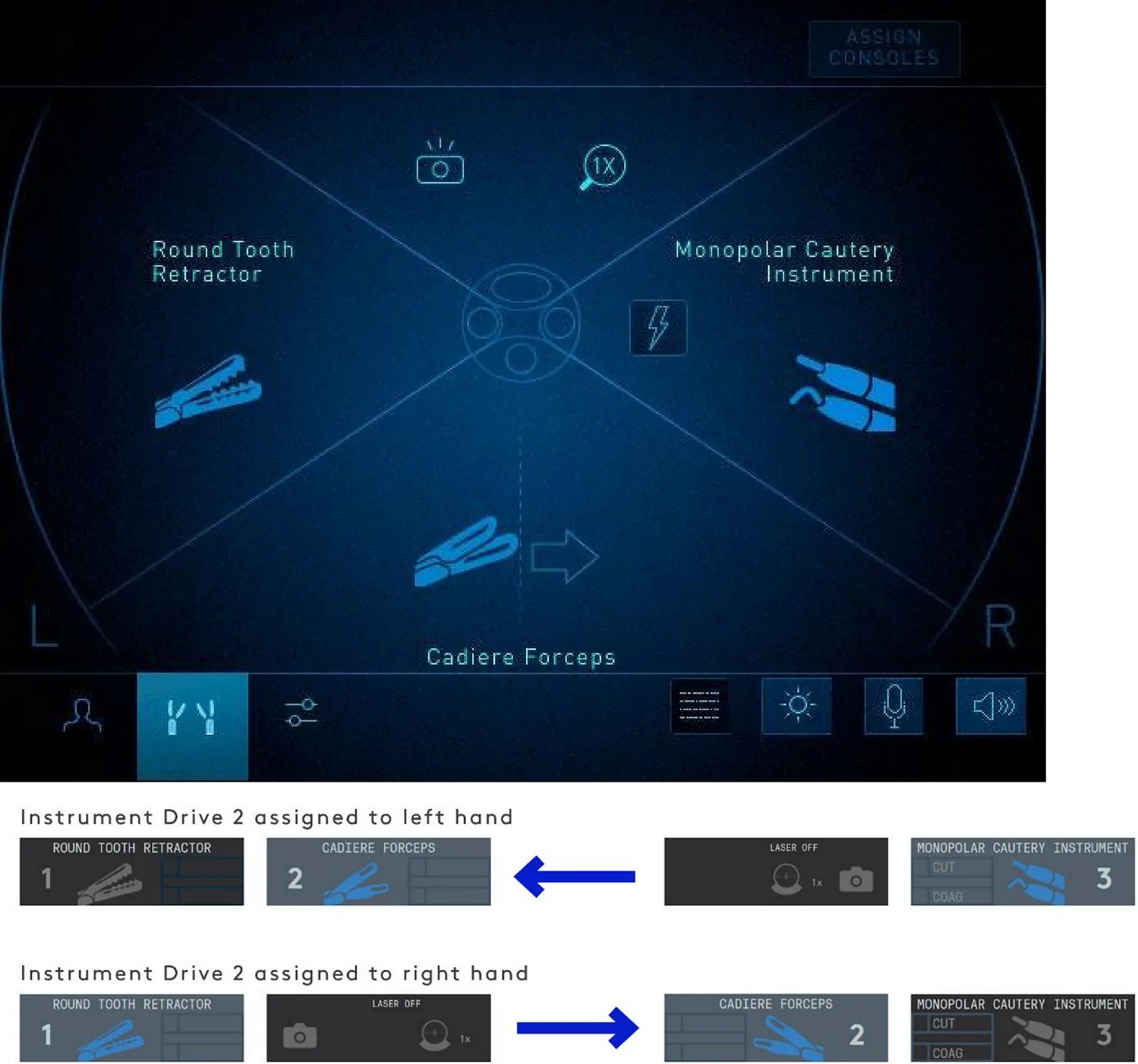
Instrument drive 2 is easily reassigned to either arm

The instrument arm contains three instrument drives each controlling a fully articulated EndoWrist instrument positioned around a central camera drive. A distinctive feature is the re-assignable Instrument Drive 2, which permits reassignment of instrument control between the surgeon’s left and right hands without requiring system undocking (Fig. 3) [15, 16]. This provides enhanced flexibility during complex dissections where instrument roles may change and allows for seamless switching between blunt and sharp dissection [19].

The surgeon console maintains ergonomic advantages of traditional da Vinci systems while accommodating SP control complexity. The console incorporates motion scaling, tremor filtration, and wristed instrument control to enable precise manipulation within confined operative fields [20]. The console interface includes specialized controls for camera articulation and instrument drive reassignment. Dual consoles are available for training and collaboration. The system’s mechanical architecture and software constraints are designed to reduce external arm collisions and internal instrument conflicts, which is particularly relevant in narrow working spaces [20]. This is most useful in transanal SP technical applications.

### Single-port robotic instruments and camera

The EndoWrist SP instruments are specifically designed for single-port applications, featuring enhanced articulation with both elbow and wrist joints, providing seven degrees of freedom for precise manipulation [15, 18]. Full list of instruments include the following:

1. Monopolar cautery
  1. Monopolar curved scissors
  2. Monopolar cautery hook
  3. Monopolar cautery spatula
2. Bipolar cautery
  1. Maryland bipolar forceps
  2. Fenestrated bipolar forceps
3. Needle driving—needle driver
4. Clip applying—medium-large clip applier
5. Grasping—cadiere forceps
6. Retracting—round tooth retractor

The SP camera system features a 12 mm 3D high-definition flexible endoscope [21] with multiple articulation points, incorporating both elbow and wrist joints [22]. The high-definition 3D vision system provides stereoscopic visualization through the surgeon console, with the ability for fluorescence imaging. This design optimizes visualization while maintaining adequate instrument working space and addressing primary challenges in SP surgery [23]. The flexible design allows navigation around anatomical structures and provides viewing angles not achievable with traditional rigid endoscopes.

### Port placement and surgical site access

For transabdominal applications, port placement typically utilizes a 3–4 cm incision (to accommodate the 25 mm cannula with adequate working space) with location dependent on procedure.

**Fig. 4:**
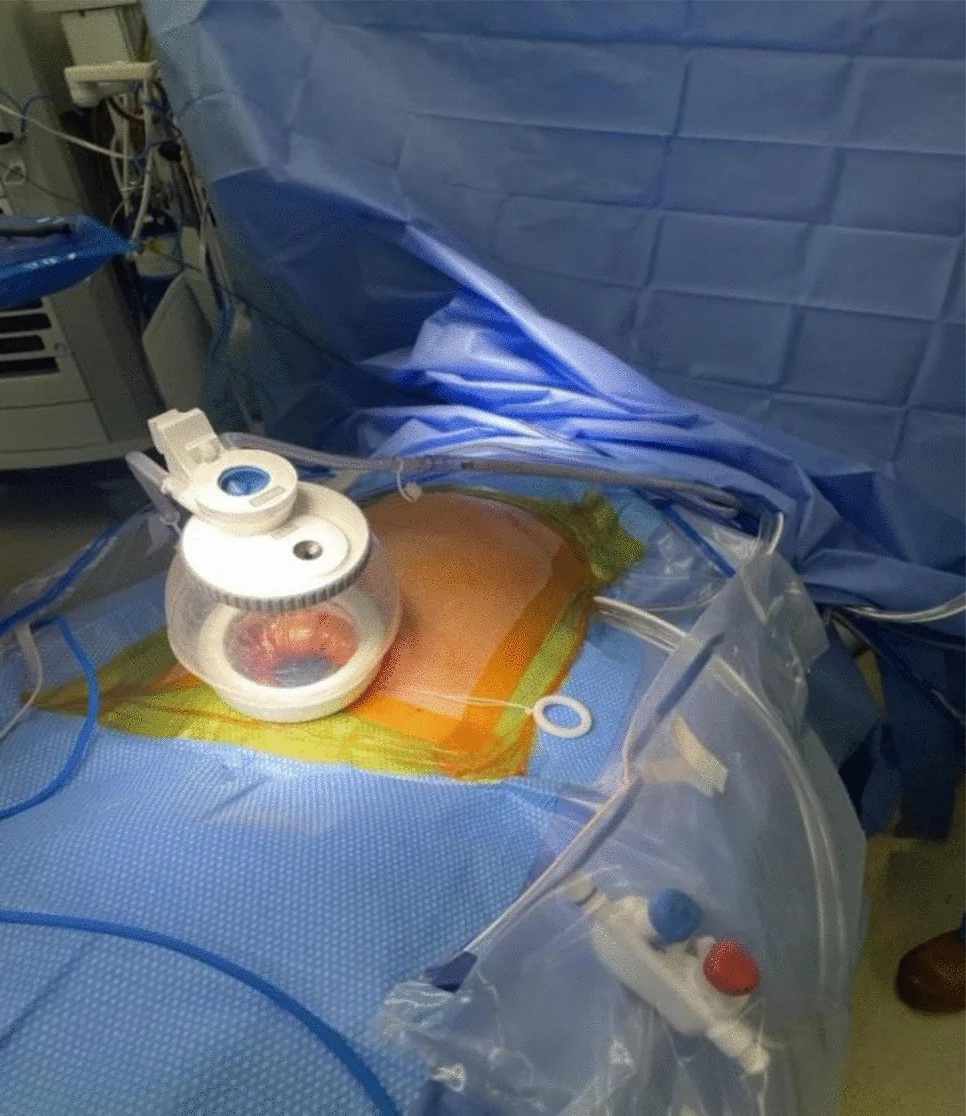
SP access port kit

**Fig. 5:**
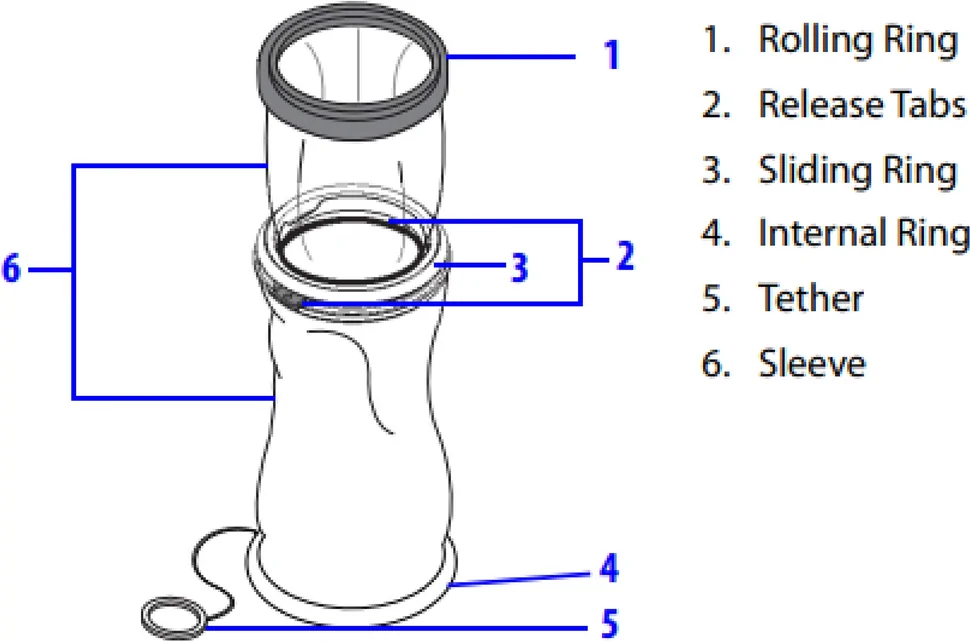
SP access port kit—wound retractor components

**Fig. 6:**
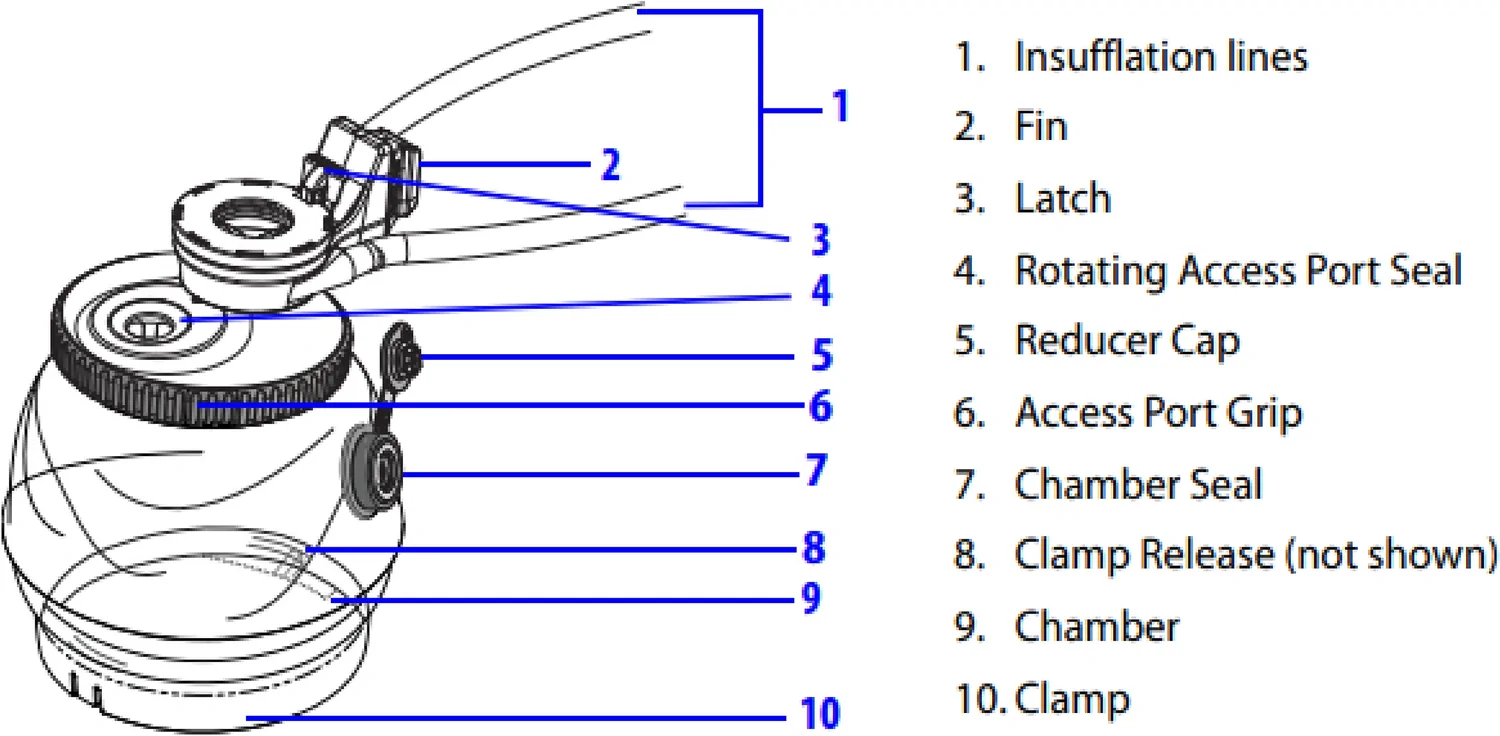
SP access port kit—access port chamber components

The SP Access Kit (Intuitive) is used for abdominal access while maintaining seal and offering free-floating docking (Fig. 4). The kit includes two parts: a wound protector which keeps the incision open with ring for connection (Fig. 5) and an access port chamber to allow free-floating docking (Fig. 6).

**Fig. 7:**
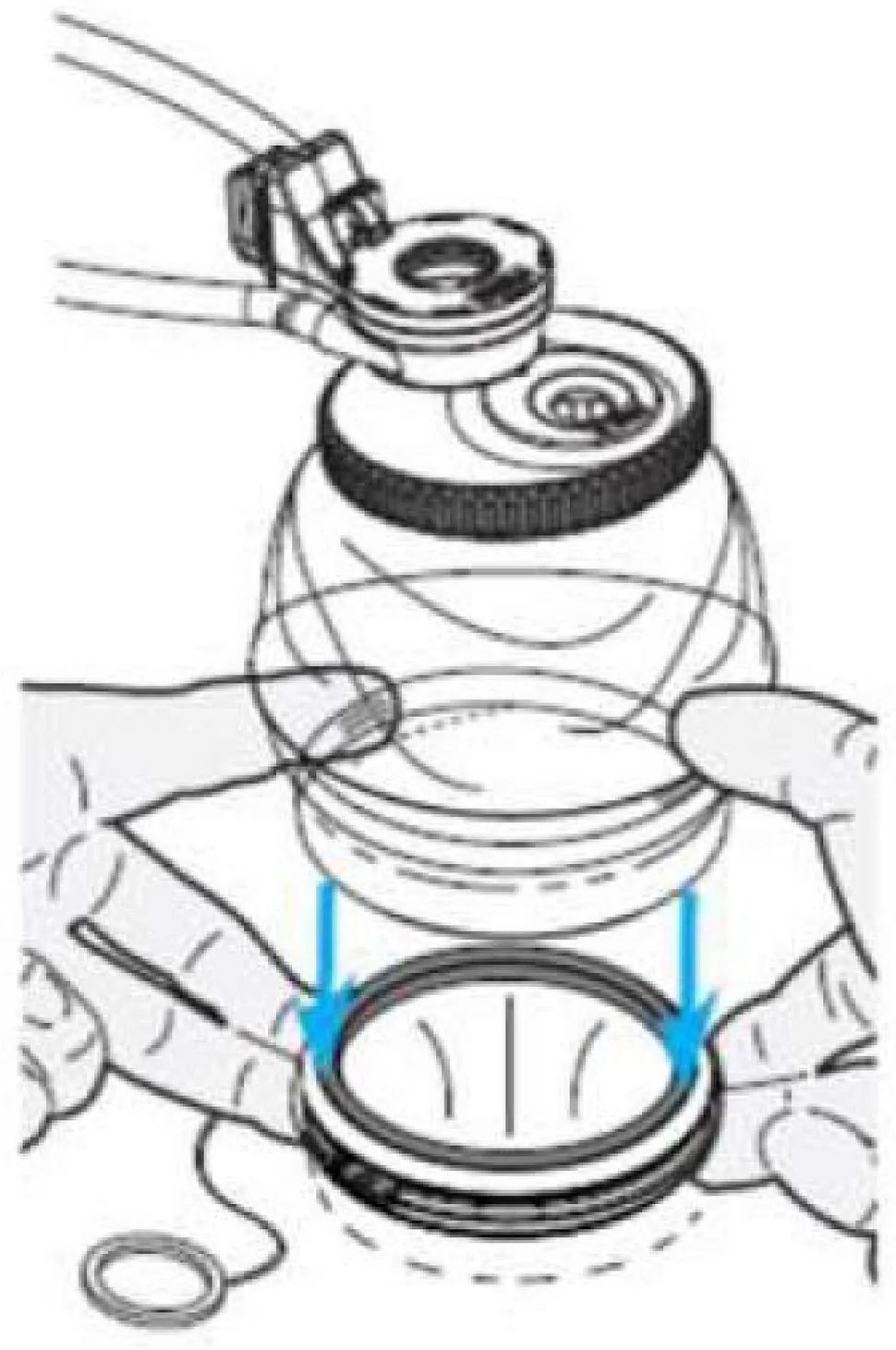
Connecting access port chamber to wound retractor

Abdominal access port placement steps:

1. An approximately 3–4 cm incision and the abdominal cavity is entered.
2. The internal ring (white) of the wound protector is placed inside the abdominal cavity after confirming no adhesions.
3. Perform finger sweep to ensure no bowel/omentum caught between ring and abdominal wall.
4. The rolling ring (Gray) is pulled up on some tension and held in place, while the sliding ring is pushed down until desired incision retraction is achieved.
5. The rolling ring is then rolled inward until the excess sleeve is reduced and is secured to the sliding ring.
6. The access port clamp is then attached to the wound retractor ensuring that the fin of the access port is pointing away from the surgical site. Two clicks should be heard to ensure secure attachment (Fig. 7).
7. Insufflate the abdomen and chamber using insufflation lines or AirSeal port to desired CO_2_ pressure.
8. Place the SP short entry guide into the access port and inspect the abdomen with SP camera.

After insufflation and camera insertion, the robot is docked with the cannula mount attached to the access port fin. Custom Remote Center selection at the console minimizes instrument-body wall collisions during the procedure.

Transanal access port placement steps:

For transanal applications, the SP cannula can be deployed through multiple access platforms. The GelPOINTTM Path Transanal Access Platform (Applied Medical, Rancho Santa Margarita, CA, USA) is the most commonly reported access platform for SP-TAMIS in published clinical series [13].

**Fig. 8:**
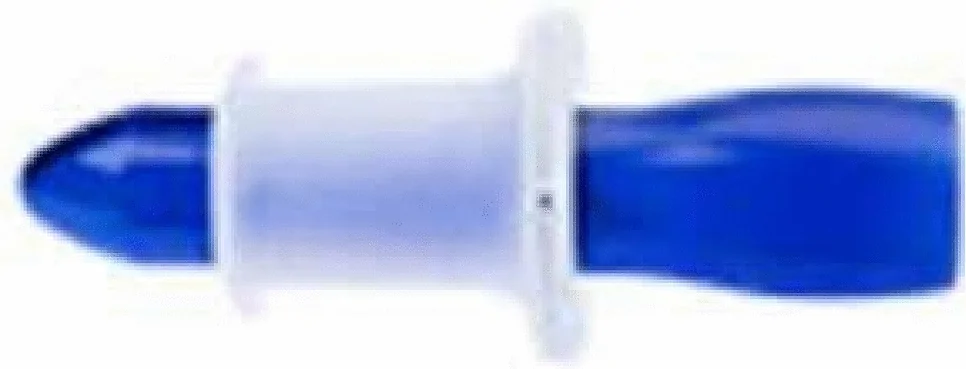
Applied medical transanal access port

The following instructions use the SP cannula with the GelPOINT Path Access Port. GelPOINT Path has two main components: The Access Channel (Fig. 8) and the GelSeal Cap.

**Fig. 9:**
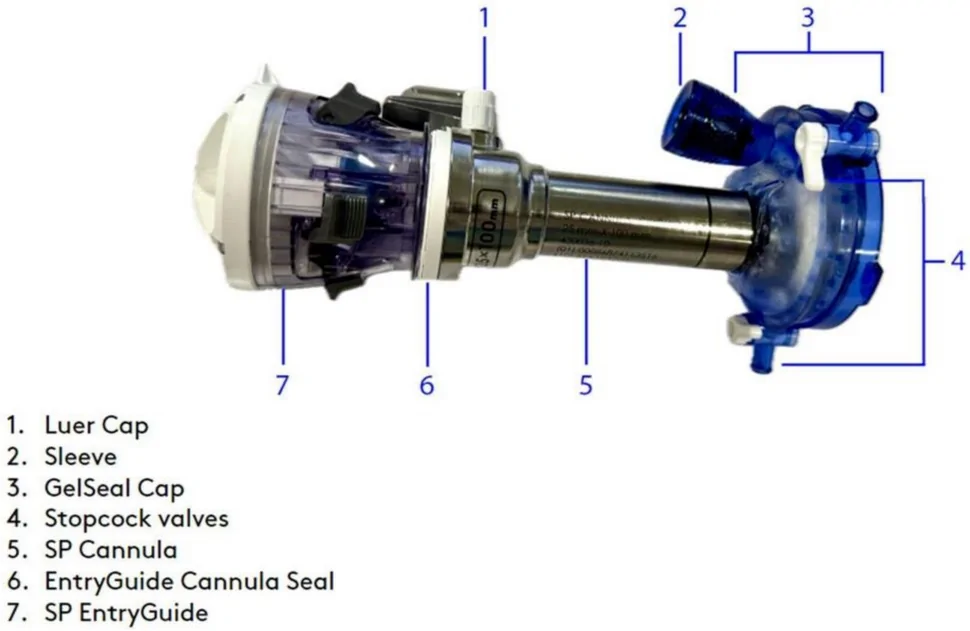
SP cannula and entry guide positioned in the GelPortPath™ Cap

1. Position the Access Channel transanally.
2. Secure the transanal access platform with sutures at the 3, 6, 9, and 12 o’clock positions to provide stable positioning while maintaining an airtight seal.
3. Place the SP cannula and obturator through the GelSeal Cap, locating the thick black ring within the cap (Fig. 9).
4. Orient the cannula fin so it points up to the ceiling and attach the GelSeal Cap to the Access Channel.
5. Optional 5- or 8-mm AirSeal® (ConMed, Utica, NY, USA) trocar is placed laterally for insufflation and smoke evacuation [24].
6. Remove the obturator and insert the SP Entry Guide.
7. Insufflate CO_2_ to a pressure of 15 mmHg to either the SP cannula or GelSeal Cap.
8. Prior to docking, the SP endoscope can be used to inspect the anatomy.

**Fig. 10:**
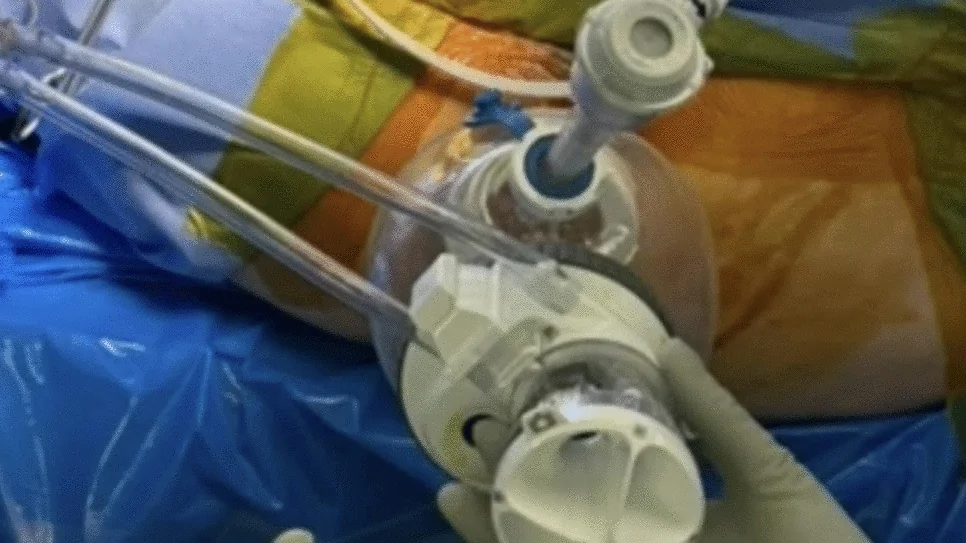
SP transanal access port

Alternative options include the da Vinci SP Small Access Port Kit (Fig. 10) or the SP cannula with compatible third-party access ports, providing flexibility based on institutional preference and availability.

**Fig. 11:**
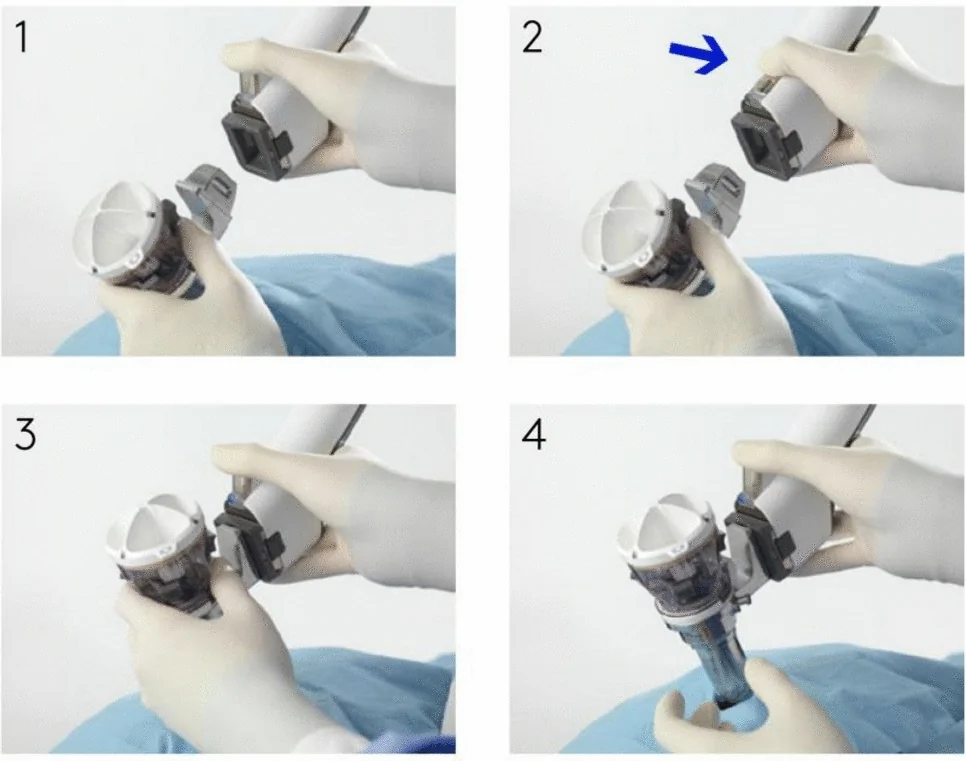
Docking SP cannula to instrument arm

Once access to the surgical site is achieved and the SP cannula is in place, the instrument arm is docked to the SP cannula with the cannula fin aimed toward the target anatomy. It is advisable to relieve the tension at the SP port site by port clutching the instrument arm and repositioning the cannula as needed (Fig. 11).

### Intraoperative controls

There are three control modes: Adjust, Camera Control, and Relocate.

**Fig. 12:**
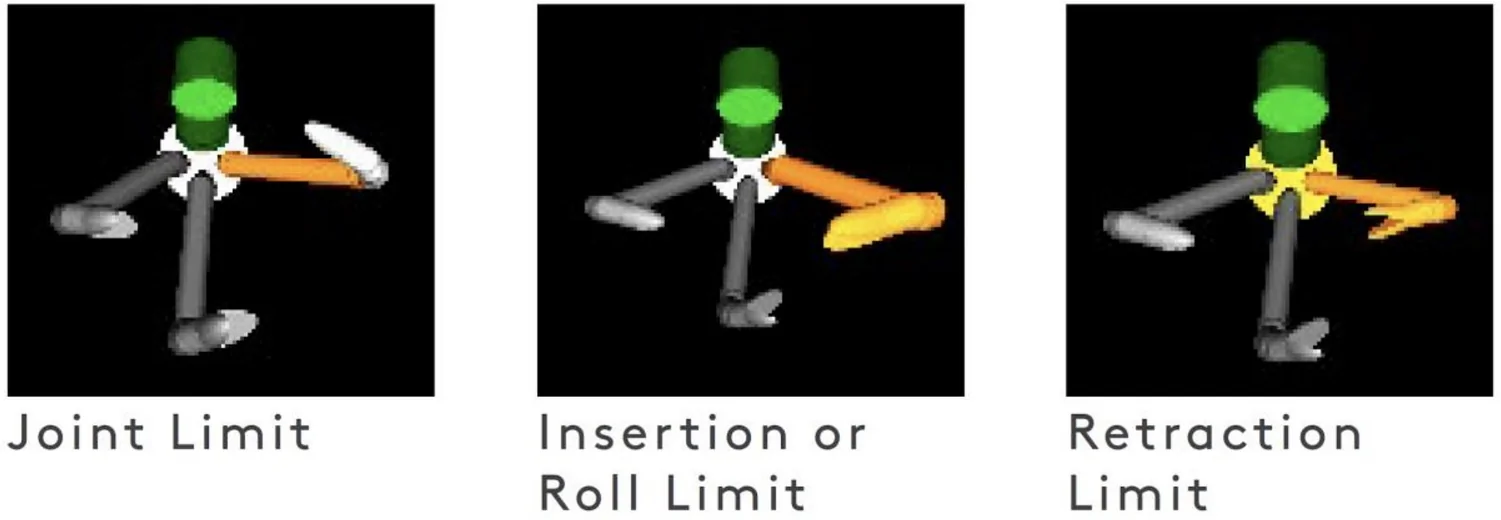
Navigator showing instrument range of motion limits

*Adjust mode* enables the surgeon to shift the workspace, while the instrument tips remain in place and the camera pose is maintained. This mode is useful for making small adjustments to the workspace. When an instrument or instrument joint reaches a range of motion limit, Adjust mode will be restricted in one direction. The instrument or instrument joint at a limit can be identified using the navigator (Fig. 12).

**Fig. 13:**
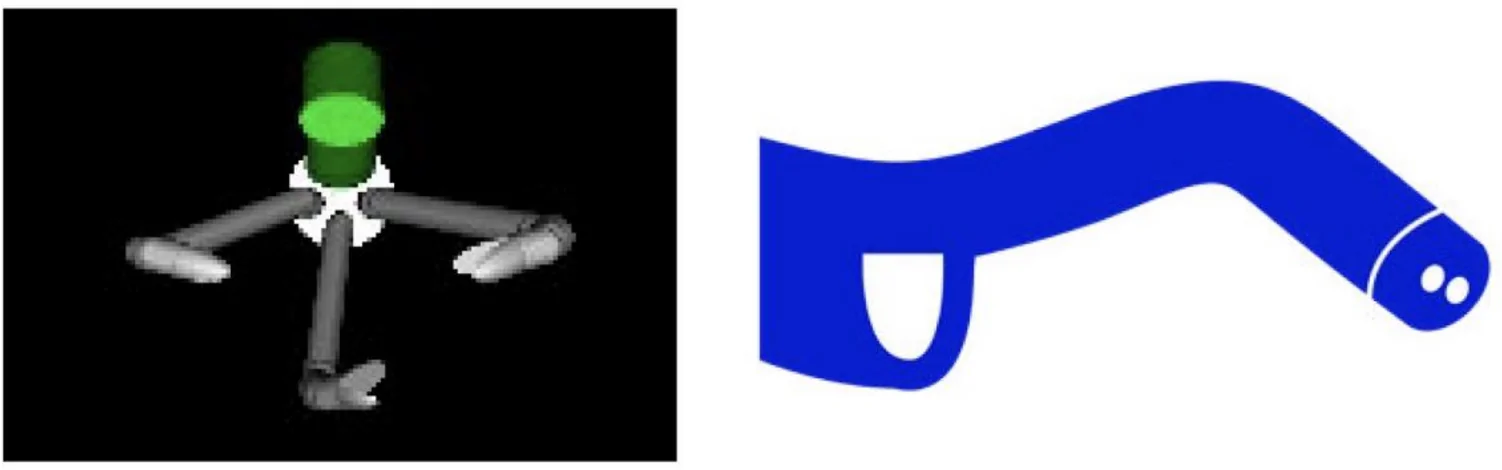
Cobra position of the flexible endoscope

*Camera Control mode* enables the surgeon to change the pose of the camera. However, in Camera Control mode, shifting the position of the hand control bends the camera elbow joint and camera wrist. Camera Control mode can be used to position the camera in cobra position. This is done by lifting the surgeon’s hand up to raise the camera above the instruments, center the camera over the instruments, and bending the surgeon’s wrist down to view the workspace. The *cobra position* represents an optimal camera configuration achieved by lifting the camera above the instruments, centering it, and bending the wrist down to view the workspace (Fig. 13). This configuration positions the endoscope above the instruments to improve the viewing angle and reduce instrument–camera interference, which can be helpful during suturing and fine dissection. [17].

*Relocate mode* moves the instruments and camera together. This mode is useful for making large changes to the workspace.

### Camera above/below

**Fig. 14:**
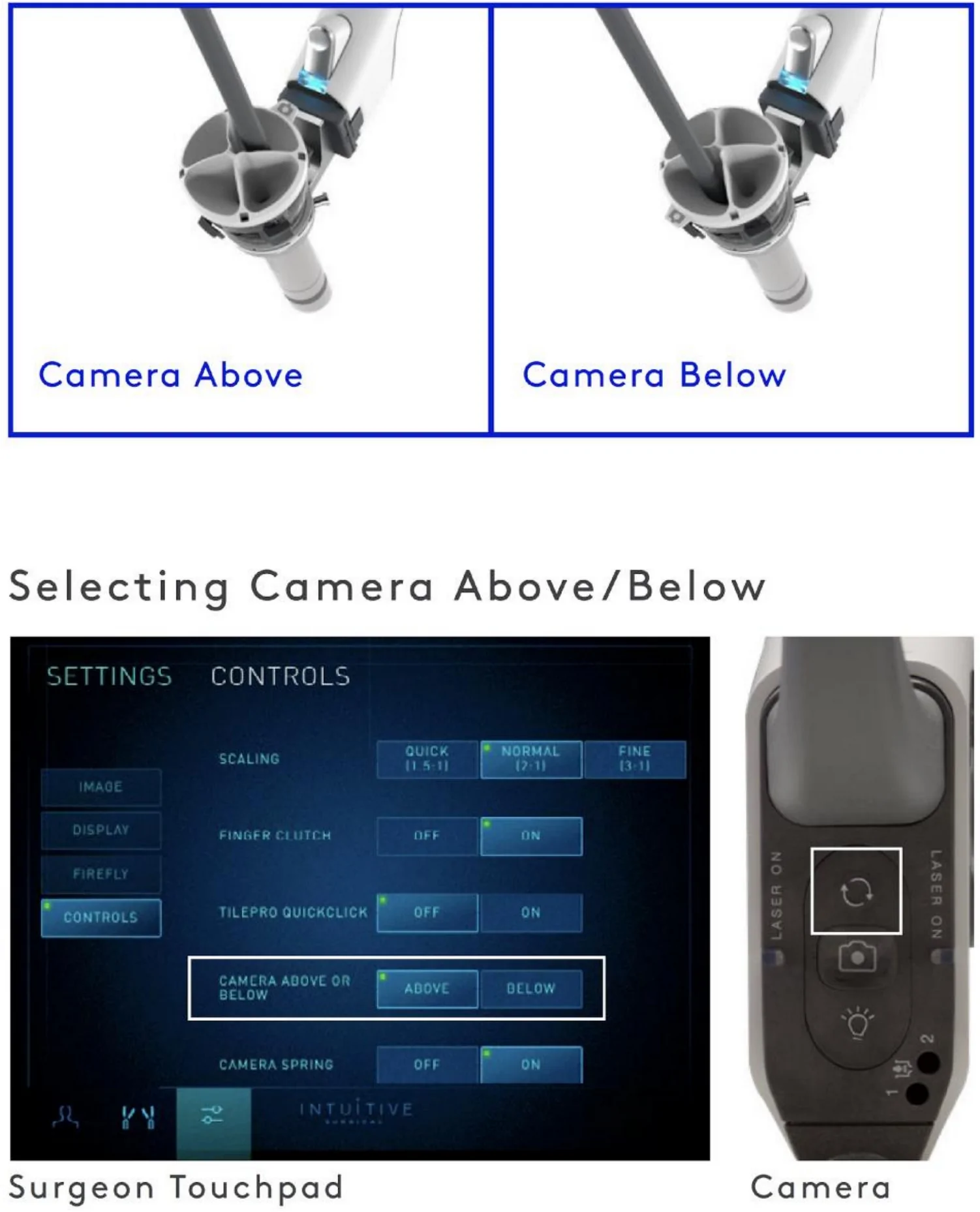
Selecting camera above/below

The system offers Camera Above (default) and Camera Below configurations (Fig. 14); in some anterior pelvic dissection scenarios, a Camera Below setup may improve the line of sight and reduce instrument–camera crowding [25]. When working anteriorly, Camera Below places instruments nearest the anatomy and provides better vantage points, which is advantageous during anterior rectal dissection where instrument–camera interference can limit visualization.

To switch between camera above/below

- If instruments are already installed, retract instruments.
- Using the arm clutch, roll the instrument drives to position the camera drive in either the above (12 o’clock) or below (6 o’clock) position.
- If the camera and instruments are not installed, roll the Entry Guide to align the camera lumen with the camera drive.
- Select corresponding setting (Camera Above or Camera Below) either from the Surgeon Console touchpad or by pressing and holding the Camera Above/Below button on the camera housing.
- If instruments were previously installed, remove and exchange instruments in Instrument Drives 1 and 3 to resume prior instrument associations.
- Console surgeon will be prompted to press the Swap pedal to confirm Camera Above/Below switch before instrument control is available.

### Training and implementation

The training pathway for SP robotics follows a structured four-phase program provided by Intuitive Surgical (Table 1) with additional procedure-specific recommendation (Table 2). For individual surgeons, recommended progression after training ideally involves starting with SP- TAMIS (Part II) to develop fundamental familiarity with the SP platform, followed by SP right colectomy (Part III) to gain experience with multi-quadrant dissection, vascular control, and intracorporeal anastomosis. Advancement to SP-LAR (Part IV) should occur only after consistent proficiency and confidence are demonstrated in these foundational techniques.

**Table 1:** Da Vinci single-port (SP) robotic training and implementation pathway

Training phase	Components	Requirements/activities	Duration/frequency
Phase I: Introduction to new technology	Initial exposure	Test drive completion Review procedure-relevant videos Live epicenter observation Standard case observation	Variable (self-paced)
Phase II: Technology training	Formal training session	Prerequisites: Technology online training Technology in-service Technology online assessment Technology skills practice (Skills Drills and SimulatorTM) Review 2 full-length procedure videos Confirm preparation for training Training: 2 day intensive training at Intuitive training center	2 days at training center (plus prerequisite time)
Phase III: Initial case series plan	Clinical implementation	Part 1: Case series Minimum 1 case per week 12–14 week implementation period Proctor assistance for initial cases Part 2: Skills development (2 activities/week): Assist in procedures Perform procedures Technology skills drills sessions Skills simulator sessions Procedure video review	12–14 weeks; 2 activities per week throughout Phase III
Phase IV: Continuing development	Ongoing education	Required: Peer-to-peer procedure training courses Additional activities (minimum 2): Physician lecture programs Complex procedure observation Complex procedure video review Webinars Peer-to-peer mentoring	Ongoing

**Table 2:** Procedure-specific recommendations

Procedure	Recommended prior experience	Initial case selection
SP-TAMIS	Conventional TAMIS or TEM Basic laparoscopic skills	Small posterior mid-rectal benign lesions Progress to larger/anterior lesions
SP right colectomy	SP-TAMIS proficiency Laparoscopic/robotic colectomy	Straightforward ileocecectomy Progress to extended right hemicolectomy
SP-LAR	SP-TAMIS proficiency SP right colectomy experience Multiport robotic LAR	Upper rectal lesions Favorable anatomy Progress to mid/low rectal cancers

Key training and implementation considerations include the following:

- Institutional Support: Ensure administrative backing for longer operative times during learning curve.
- Team Training: Entire operative team requires familiarity with SP equipment and troubleshooting.
- Bedside Assistant Skills: Critical for energy device and stapler management (importance increases with procedure complexity).
- Case Volume: At least one case per week recommended to maintain skill progression.
- Proctoring: Important for initial cases in each procedure category.

## Conclusion

This manuscript details the important technical elements of the currently FDA-approved SP robotic platform including the fundamental tools, techniques, and training pathways that are essential to start a Colorectal Surgery SP program. This manuscript serves as a comprehensive overview and reference document for a four-part series detailing SP implementation into three specific colorectal surgical procedures. The progression of SP implementation from foundational SP-TAMIS, through the intermediate complexity of SP right colectomy, to advanced pelvic procedures such as SP-LAR promotes structured skill acquisition. This systematic approach allows surgeons and institutions to expand capabilities safely while ensuring consistent and high quality surgical outcomes.

The Society of American Gastrointestinal and Endoscopic Surgeons (SAGES) Colorectal Surgery SP Task Force Subject Matter Experts recommend structured, evidence-based adoption of new technologies, emphasizing patient safety, quality of care, and continuous outcome monitoring [26]. This paper is limited in that its recommendations are based on expert opinion rather than high-level evidence. Currently, published data on SP colorectal surgery remain limited to clinical experience and small case series, highlighting the need for larger, prospective studies to further validate these findings. Several future research priorities remain to further advance the understanding of SP robotic colorectal surgery, including prospective comparative studies, standardized learning curve assessments, evaluation of patient-reported outcomes, and cost-effectiveness analyses. Surgeons are encouraged to pursue formal SP robotic training, simulation, and proctoring, while institutions are encouraged to ensure appropriate resources and team readiness training.

Future advances, including a wider range of SP platforms, SP-specific staplers, energy devices, and standardized training curricula, will further enhance procedural efficiency and safety. Multicenter collaboration and simulation-based education will be essential to shorten the learning curve and promote responsible dissemination. As experience accumulates and costs decrease, SP robotic surgery may evolve from a specialized innovation to a standard minimally invasive option for selected colorectal patients.
